# Improving Vaccine Knowledge Among Adolescents: A Pre–Post School-Based Educational Intervention in Southern Italy

**DOI:** 10.3390/vaccines14020153

**Published:** 2026-02-04

**Authors:** Vincenza Sansone, Gaia D’Antonio, Grazia Miraglia del Giudice, Francesco Napolitano, Gabriella Di Giuseppe

**Affiliations:** 1 Department of Experimental Medicine, University of Campania “Luigi Vanvitelli”, Via Luciano Armanni 5, 80138 Naples, Italy; 2 Department of Public Health and Laboratory Services, Teaching Hospital of the University of Campania “Luigi Vanvitelli”, Via Luciano Armanni 5, 80138 Naples, Italy

**Keywords:** adolescents, knowledge, school-based educational intervention, vaccine literacy, vaccination

## Abstract

**Background/Objectives**: Vaccination coverage among adolescents remains below the recommended target, highlighting the need for effective educational strategies to improve vaccine knowledge. This study aimed to assess baseline knowledge of vaccines and immune mechanisms among adolescents and to evaluate whether a school-based educational intervention can improve knowledge related to vaccination. **Methods**: A prospective quasi-experimental pre–post study was conducted between 1 February 2025 and 1 June 2025 among adolescents aged 14–19 years attending high schools in Southern Italy. The intervention was based on the e-Bug educational module and delivered by trained nurses through interactive lessons, gamification, and guided discussions. Vaccine-related knowledge was assessed using a questionnaire administered before and after the intervention. Changes in knowledge scores were analyzed using paired statistical tests, and the effect size was estimated. A stepwise multivariate linear regression model was employed to identify factors associated with post-intervention test scores, with statistical significance set as *p* ≤ 0.05. **Results**: Among 386 participants, the majority were female (74.2%), the average age was 15.8, and 15% reported a chronic medical condition. Knowledge gaps were observed at baseline, particularly regarding the items on recommended adolescent vaccinations (37.4%), the definition of innate immunity (25.6%), and the mechanism of vaccines’ action (51%). After the intervention, all the items showed an improvement in correct answers, statistically significant for 5 of the 7 analyzed items (r = 0.364, *p* < 0.001). The most pronounced improvement was in the awareness of age-specific recommended vaccines (61.2%). The multivariate linear regression analysis showed that those with higher pre-intervention test scores, those who had parents with chronic medical conditions, those whose fathers worked, and those willing to participate in similar future interventions were more likely to achieve higher post-intervention test scores. **Conclusions**: School-based interventions may represent an effective strategy for enhancing adolescents’ knowledge related to vaccination, but further studies with control groups and long-term follow-up are needed to confirm effectiveness.

## 1. Introduction

Vaccination is one of the most cost-effective and impactful public health strategies available, averting an estimated 3.5 to 5 million deaths each year from vaccine-preventable diseases (VPDs), including diphtheria, tetanus, pertussis, influenza, and measles [1,2,3]. Beyond providing individual protection, vaccines foster herd immunity, reducing pathogen transmission and safeguarding vulnerable populations who cannot be vaccinated [4]. 

Despite these documented advantages, adolescents represent one of the underserved populations facing barriers to adequate immunization [5].

Adolescents require specific attention, as the adaptive immunity provided by childhood vaccination decreases over time [6]. According to the Italian National Prevention Vaccination Plan, adolescents are required to receive mandatory booster doses of diphtheria, tetanus, acellular pertussis, and inactivated polio vaccine (dTaP-IPV), as well as age-specific recommended vaccines such as meningococcal serogroup ACWY (MenACWY) and Human Papillomavirus (HPV) vaccines [7,8]. Nevertheless, vaccination coverage in Italy remains below the optimal target. Recent data, presented by the Italian Ministry of Health, show that the coverage at 16 years of age remains well below the ≥95% target, with approximately 69% completing dTaP-IPV, 57% receiving MenACWY, and 58–68% finalising HPV vaccination, with differences between genders [9,10].

Moreover, adolescence is also characterized by increased socialization, which intensifies exposure to infectious agents [11], alongside exploratory risky behaviors that can establish negative health patterns persisting into adulthood [12]. Traditionally, vaccination decisions are made by parents, but the COVID-19 pandemic intensified vaccine hesitancy among adults, with distrust extending even to established childhood immunization schedules [13,14,15]. Simultaneously, European data show vaccine hesitancy prevalences of 12.5%, 14%, 24.7%, and 31.6% among adolescents in Spain, Portugal, Italy, and Poland, respectively [16]. Moreover, adolescents are increasingly asserting their desire to participate in health decisions, including those regarding vaccination [17], and request accessible, trustworthy information to support their involvement [18,19]. In this context, vaccine literacy represents a specific domain of health literacy encompassing the knowledge, motivation, and competencies to access, understand, appraise, and apply information about immunization [20,21]. This construct includes functional literacy related to basic vaccine knowledge, such as how vaccines work and their benefits and risks, interactive literacy involving the ability to seek and apply information from different sources to one’s personal health context, and critical literacy, which refers to analyzing information credibility, identifying misinformation, and making autonomous evidence-based vaccination decisions [20].

However, evidence consistently shows that many adolescents have inadequate knowledge about vaccination and VPDs, with misconceptions that may hinder their ability to participate effectively in vaccination decisions and may compromise future vaccine uptake [17,22,23,24,25]. Schools are an important setting for health-promotion interventions enhancing adolescent knowledge, shaping motivation, and improving vaccine knowledge [26,27]. Indeed, the Health Promoting Schools (HPS) framework has demonstrated that coordinated school-based actions can positively influence knowledge and attitudes about disease prevention, including vaccination [27,28,29]. Some international evidence has examined the vaccine knowledge improvement in adolescents through a structured school-based intervention [30,31], but evidence from Italy remains limited [32,33]. Despite the availability of recommended adolescent vaccinations, Italy does not have a well-structured school-based immunization or vaccination education program, and school health promotion activities are mainly implemented on a voluntary and project-based basis by Local Health Authorities, leaving many students with limited access to trustworthy vaccination information [32]. Therefore, the present study aims to assess adolescents’ knowledge of vaccines and immune mechanisms, and to evaluate whether a school-based pre-post intervention can improve knowledge related to vaccination.

## 2. Materials and Methods

### 2.1. Setting and Participants

A prospective quasi-experimental pre-post study was conducted in the Campania Region, South of Italy, from February to June 2025 among a sample of adolescents aged from 14 to 19 selected through a two-stage cluster sampling. In particular, from the list of public schools in the region, four high schools were randomly chosen, and, in each school, 7 classes were randomly selected to be involved in the study. In Italy, public high schools represent the primary educational environment for adolescents aged 14–19 years and enrol a large majority of this age group, thus providing access to a socioeconomically and culturally diverse sample. The following inclusion criteria were applied: adolescents aged 14–19 years; parental consent for participants younger than 18 years; personal informed consent for participants aged 18 years or older; and absence of teacher-reported disabilities.

### 2.2. Data Collection

To involve the selected schools in the data collection, the deans were approached to plan an introductory meeting with a member of the research team to explain the study aims and the procedures for data collection. After the dean accepted, each adolescent was handed an envelope containing a cover letter, which described the study’s purpose and provided information on how to participate, the measures to guarantee the anonymity and confidentiality of the data collection, and a written informed consent form. The cover letter clarified that the intervention was offered to all adolescents (and therefore no non-intervention/control group was included) and underlined that participation was voluntary. The adolescents were invited to give the envelope to one of their parents and to return the signed informed consent. According to the school’s dean, a team member returned ten days later to collect the completed consent forms. No payment or contributions would be given to participants. Adolescents aged 18 years or older provided their own consent to participate. For respondents under 18 years, parental consent as well as individual assent were required to participate. This approach ensured an appropriate balance between parental consent and adolescent autonomy in health decisions. The educational intervention was conducted in person, either in the classroom or in the common areas of the school. At the beginning of the intervention, each student was provided with a self-administered and paper-based questionnaire (Supplementary Materials) to complete individually. Approximately 10 min were required to complete the questionnaires, which were collected by the research team immediately after the intervention for subsequent data analysis. 

The pre–post design study without a control group was chosen to offer the intervention to all eligible adolescents and avoid withholding a potentially beneficial educational program. The questionnaire was tested with a pilot study among 50 adolescents to confirm clarity, feasibility, and to estimate the standardized effect size (Cohen’s d = 0.44), corresponding to a medium effect [34]. Using this estimate for an a priori power analysis with α = 0.05 (two-tailed) and 80% power, the calculated sample size required to achieve the desired precision was 43 participants. As no modifications were made to the study protocol following the pilot study, data from the pilot study participants were included in the final analysis. The final study sample comprised 386 participants, representing all students in the selected classes who provided consent to participate. The larger sample size increases the generalizability and statistical power of the study [35].

The study protocol was approved by the Ethics Committee of the University of Campania “Luigi Vanvitelli” (prot. N.0018199/i/2024).

### 2.3. Intervention

The intervention was based on the e-Bug educational module designed for Key Stage 4 students (14–16 years) [36], adapted for the Italian context. The content focused on innate and acquired immunity, herd immunity, and vaccinations. The module comprised interactive activities, gamification, and group discussions to engage students.

A structured, self-administered questionnaire, divided into four sections, based on EBUG educational materials and adapted by the research team to the Italian context [36], was used for data collection. The first section collected sociodemographic data (such as age, gender, number of cohabitants, etc.). The second and third sections assessed knowledge and were administered twice: before the intervention (pre-test, second section) to establish baseline knowledge, and immediately after the intervention (post-test, third section) to measure the changes in knowledge. Knowledge was evaluated using 7 multiple-choice questions to investigate the functioning of the immune system, the mechanism of action of vaccines, the concept of herd immunity, and knowledge on the recommended vaccines for participants’ age group. Each question had four answer options, only one of which was correct, and required the participants to select the one they deemed most appropriate.

The last section of the questionnaire was dedicated to the subjective perception of the intervention. The first three questions used a 5-point Likert scale (from 1 = “not at all” to 5 = “completely”) to evaluate the overall level of satisfaction with the educational intervention, the perceived clarity of the information provided, and the perceived usefulness of acquired knowledge in daily health choices. The fourth question explored the need for further information on vaccination, and the final question assessed adolescents’ willingness to participate in future interventions and identified their topics of interest.

The intervention was carried out in classrooms or common areas (school gyms or theatres) by trained nurses who had received a one-month methodological training based on e-bug materials [36]. At least three researchers were present in each session to teach, monitor, conduct game play and discussion, and collect the completed questionnaires. The educational intervention consisted of a 50-min lesson with slides built according to the e-bug project instructions, with graphics that would keep students’ attention. Additionally, a group game using playing cards was organized in order to simulate herd immunity. Finally, a group discussion focused on common vaccine misconceptions was guided by the researchers.

The educational intervention concluded with a summary slide containing the take-home messages, and each student received a printed QR code linking to an illustrated brochure for future reading and sharing with relatives and friends.

### 2.4. Statistical Analysis

Data were entered and analyzed using STATA 17. Descriptive statistics, including frequencies and percentages, were used to summarize sociodemographic characteristics and the proportions of correct/incorrect answers for each knowledge question in both pre- and post-test.

The outcome measures were two scores obtained by summing the number of correct responses on the test, assessed at two time points: knowledge score before the intervention (“pre-test”—outcome 1) and knowledge score after the intervention (“post-test”—outcome 2). Scores were treated as continuous variables, and means and standard deviations were computed for both pre- and post-test scores.

To assess whether the differences in scores met the assumption of normality required for parametric analyses, the Shapiro–Wilk test was used. A value of *p* < 0.05 indicated a deviation from normality, justifying the use of non-parametric methods. Because the Shapiro–Wilk test indicated a significant departure from normality in the difference scores, the non-parametric Wilcoxon signed-rank test was used to compare pre- and post-intervention scores. The Wilcoxon signed-rank test examines whether the median of the differences was significantly different from zero. The threshold for statistical significance was set at α = 0.05, two-tailed.

Although the distribution of the differences between pre- and post-intervention scores deviated from normality, a paired *t*-test was performed to compare the measurements. This approach was considered appropriate because the sample size was sufficiently large, and the paired *t*-test is generally robust to moderate violations of the normality assumption in continuous paired data [37,38].

The magnitude of the intervention effect was quantified by computing the effect size r. McNemar’s test for dichotomous items (correct/incorrect) was used to compare the changes in knowledge between pre- and post-intervention assessments.

A stepwise multivariate linear regression model was performed to identify the determinants of knowledge score after the intervention (outcome 2, continuous). The model was developed according to Hosmer and Lemeshow’s model-building strategy [39], which includes the bivariate analysis of each independent variable, and those with a value of *p* ≤ 0.25 were included in the final model. The values for variables’ entry and removal in the final models were, respectively, *p* = 0.2 and *p* = 0.4 throughout the stepwise selection procedure. The following independent variables have been tested: age (continuous); gender (female = 0; male = 1); nationality (foreign = 0; Italian = 1); number of cohabitants (continuous); type of school (Higher Technical Institutes = 0; high schools = 1); having the father employed (no = 0; yes = 1); having parents with chronic medical conditions (no = 0; yes = 1); being satisfied with the educational intervention (not at all/slightly/moderately/very = 0; completely = 1); considering the information provided as clear (not at all/slightly/moderately/very = 0; completely = 1); willingness to participate in similar future interventions (no = 0; yes = 1); and the pre-intervention test scores (continuous). Results are presented as *β* coefficients. All reported *p*-values are two-tailed, and a *p*-value ≤ 0.05 is estimated as statistically significant. Missing data regarding socio-demographic and anamnestic characteristics were addressed using a complete-case analysis, and participants with missing values were excluded from the analysis, whereas missing values for the knowledge score were treated as “incorrect” responses.

## 3. Results

### 3.1. Characteristics of the Participants

Among the 635 adolescents selected, a total of 386 provided consent to participate, yielding a response rate of 60.7%. The principal characteristics of the sample are presented in Table 1. A large majority of participants were female (74.2%) and Italian (94.2%), the average age was 15.8 years, the average number of cohabitants was 3.6, and 15% of participants reported having a chronic medical condition. Regarding their parents, 5.2% had both parents with a university degree, 34.2% had both parents employed, and 20.9% had a parent with a chronic medical condition.

### 3.2. Knowledge Related to Vaccination

Participants’ knowledge of vaccination and the immune system was assessed before the intervention. Overall, 89.6% of participants were aware of what the immune system is, and 25.6% and 58% recognized innate and naturally acquired immunity, respectively. Regarding knowledge on vaccinations, 51% of participants were aware of how vaccines function, 61.2% knew their advantages, and 72.5% knew the meaning of herd immunity. Lastly, only 37.4% of participants were aware of all the vaccines recommended for their age group (Table 2), whereas 43.7%, 11.8%, and 7.1% correctly identified only HPV, meningococcal, and dTaP-IPV vaccines, respectively.

**Figure 1:**
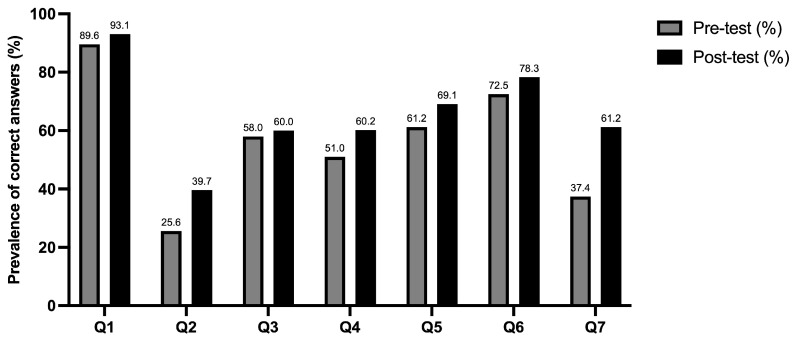
Prevalence of correct answers at pre- and post-intervention by questions.

After the intervention, all the items showed an improvement in the proportion of correct answers (Figure 1). In particular, 93.1% were aware of what the immune system is, and 39.7% and 60% recognized innate and naturally acquired immunity, respectively. Regarding vaccination knowledge, 60.2% were aware of how vaccines work, 69.1% knew their advantages, and 78.3% knew the meaning of herd immunity. Lastly, 61.2% of adolescents were aware of the vaccines recommended for their age group. The McNemar test indicated significant pre–post intervention changes for 5 of the 7 analyzed items; detailed results for all items are reported in Table 2. In particular, responses to Q1 (What is the immune system?) and Q3 (Which one of the following is an example of naturally acquired immunity?) regarding the immune system did not show statistically significant improvements.

Among all participants, the intervention led to significantly increased vaccination knowledge, with a mean pre-intervention score of 3.93 ± 1.42 and a mean post-intervention score of 4.51 ± 1.72. A subgroup analysis by gender showed that both males and females demonstrated significant improvement in knowledge following the intervention. Males showed a mean pre-intervention score of 3.94 ± 1.37 and post-intervention score of 4.33 ± 1.68, while females had a mean pre-intervention score of 3.95 ± 1.43 and post-intervention score of 4.60 ± 1.74. No statistically significant differences were observed between genders in either baseline knowledge (*p* = 0.944) or post-intervention knowledge (*p* = 0.177).

Before analysis, the normality of the difference scores was assessed using the Shapiro–Wilk test, which indicated a significant deviation from normality (*p* = 0.006). Therefore, a nonparametric Wilcoxon signed-rank test was performed to compare pre- and post-intervention scores. The analysis revealed a statistically significant increase in post-intervention scores compared to pre-intervention (Z = 7.17, *p* < 0.001). The effect size (r = 0.364) suggests a medium-to-large magnitude of change, indicating that the intervention produced a substantial improvement in participants’ performance, confirmed by the paired *t*-test (t = −6.796; *p* < 0.001). Results of the Wilcoxon signed-rank test and paired *t*-test were consistent, confirming the reliability of the findings.

### 3.3. Adolescents’ Perception of the Intervention

More than half of the participants reported complete satisfaction with the educational intervention (53.1%), with no significant difference between males (46.8%) and females (55%) (*p* = 0.170), and considered the information provided completely clear (54.2%), while 46.7% perceived the acquired knowledge as completely useful for making daily health-related decisions. Additionally, 31.2% of participants indicated a need for further information on vaccination, and 33.2% expressed willingness to participate in similar future interventions.

### 3.4. Multivariate Analysis

The multivariate linear regression model showed that those with higher pre-intervention test scores, those who had parents with chronic medical conditions, those whose fathers worked, and those willing to participate in similar future interventions were more likely to achieve higher post-intervention test scores (Model in Table 3).

## 4. Discussion

This study aimed to evaluate the impact of school-based educational intervention on improving vaccination knowledge among high-school students in Italy. To our knowledge, this is the first study conducted in this setting to assess general immunization knowledge and specific vaccination recommendations using a structured pre-post design.

Before the intervention, students showed limited understanding of essential concepts related to vaccinations. Less than two-thirds of participants recognized that vaccinations reduce the spread of VPDs. This proportion is lower than that observed among 14-year-old students in Austria, where 82.6% answered correctly [40]. Conversely, 72.5% of students correctly identified the concept of herd immunity, a substantially higher prevalence than that reported among German adolescents, where only 39% demonstrated adequate understanding [41].

Regarding the knowledge of specific adolescent-recommended vaccinations, 81.1% of the sample correctly identified the HPV vaccine as recommended during adolescence. This result is higher than what was observed in investigations conducted in Nigeria and France, where only 56.5% and 59% of students, respectively, had heard of the HPV vaccine [42,43]. Moreover, almost half of the participants identified the meningococcal vaccine as recommended for adolescents, a result consistent with previous findings among Italian adolescents [24].

The present study shows that the educational intervention improved students’ knowledge across all assessed items. Specifically, the mean knowledge score increased by 14.8%, from 3.93 to 4.51. The most notable improvement concerned the identification of vaccinations recommended for adolescents, which increased by 23.8%. These results suggest that, while basic awareness of vaccinations was present, students lacked clarity regarding specific immunization recommendations, gaps that the intervention was effective in addressing. However, given the absence of a control group, causal interpretations should be made with caution, as the observed improvements may partially reflect the influence of external factors, such as concurrent exposure to vaccination-related information through media or peer discussions. Nevertheless, since the questionnaire was administered right after the intervention, the potential influence of external factors is likely to have been minimized.

Evidence from other countries supports the effectiveness of educational interventions in improving health knowledge. For example, structured health education delivered to rural women in Bangladesh and Ghana substantially improved awareness of cervical cancer, from 69.5% [44] and 84.2% [45] at baseline, respectively, to full awareness after the intervention. Our findings are also consistent with previous research demonstrating that school-based educational activities, whether through lectures, interactive sessions, or combined approaches, significantly improve knowledge about HPV and vaccinations among both students and parents [43,46,47,48]. Similar findings have also been documented in studies exploring health education and vaccination knowledge in various settings [49,50]. The improvement observed confirms the importance of implementing similar educational strategies in school environments.

Improving vaccination knowledge is crucial since higher levels of understanding are strongly associated with greater vaccine acceptance and uptake. Several studies, indeed, have demonstrated that increased knowledge reduces hesitancy and improves willingness to be vaccinated [51,52,53,54]. Moreover, evidence from COVID-19 research shows that education-based interventions can directly increase vaccination uptake [55,56]. Such interventions are effective, probably because they provide structured, context-specific, and accessible information that addresses misconceptions and bridges knowledge gaps. Prior work shows that these approaches enhance health literacy, correct misinformation, and support informed decision-making [57].

The stepwise multivariate regression model identified several factors associated with the profile of adolescents who had higher post-intervention vaccination knowledge scores. In particular, having parents with chronic medical conditions was a significant predictor of improved knowledge following the educational intervention. A possible explanation could be that adolescents with parents affected by chronic conditions could be more frequently exposed to healthcare settings and discussions about preventive measures. This repeated exposure could improve familiarity with health-related information and help knowledge acquisition, as suggested by previous studies confirming that the family’s vaccination status and health context significantly influence vaccination attitudes and behaviors [58,59,60,61]. Indeed, although direct evidence is limited, several experiences in the literature indicated that the family is a key setting for health promotion, that familial health context influences health-related awareness, and that it can enhance adolescents’ engagement with health information and appropriate preventive behaviors [62,63]. Moreover, adolescents who expressed willingness to participate in educational future interventions were more likely to achieve higher post-intervention test scores. This result may indicate a higher level of trust in the educational process, even given that more than half of the study participants reported complete satisfaction with the proposed educational intervention. It is important to underline that the substantial unexplained variance in the Model (approximately 70%) may reflect unmeasured factors, such as prior exposure to vaccination information, differences in learning capacity or motivation, engagement during the intervention, or other individual characteristics, suggesting that future studies should explore additional variables to better understand adolescents’ responses.

Overall, 31.2% of students expressed the need for additional vaccination information, suggesting that a standardized intervention may not fully address all individualized or preferred learning needs. Future studies could incorporate qualitative feedback, for example, using focus groups or open-ended questions, to identify students’ preferred topics and delivery formats. Nevertheless, despite the overall improvement, post-intervention knowledge of several immunological concepts remained suboptimal, in particular regarding the innate and herd immunity. These findings underscore the complexity of immunology-related topics, which could require repeated reinforcement to be fully understood, and the developmental stage of the study population. Adolescence cognitive development is characterized by a gradual progression from functional health literacy, focused on foundational knowledge, toward an interactive and critical literacy, which requires higher-order evaluative skills [20]. Since the participants’ mean age was 15.8 years, full understanding of immunological concepts may be challenging, especially within the context of a short-term educational intervention. Therefore, the persistence of these knowledge gaps suggests that short-term educational interventions may be insufficient to ensure durable improvement in literacy. Evidence from school-based health promotion research indicates that effective interventions targeting child and adolescent populations should involve multiple providers and target groups through the engagement of schools, health professionals, and families, and employ a well-structured design with adequate session duration and strategically planned follow-ups to maximize knowledge retention [64]. Adequate comprehension of these topics is crucial for developing informed attitudes toward immunization and may reduce susceptibility to misinformation, which spreads enormously, especially on social media. Instead, targeted age-appropriate educational strategies about immunity and vaccination in adolescents are needed to improve long-term learning with multimodal, repeated, and developmentally tailored educational approaches. Interventions should move beyond information delivery and foster conceptual understanding, motivation, and critical thinking, thereby supporting informed health decisions throughout life.

Overall, these results confirm that educational interventions delivered in adolescent populations can significantly improve vaccine-related knowledge, especially when addressing previously identified gaps. Enhancing immunization knowledge during adolescence may contribute to more informed health behaviors and greater vaccination acceptance.

### Limitations

The interpretation of the results should take into account the possible methodological limitations inherent in any similar study. First, a single-arm pre-post design was employed, without a control group, which limits the ability to draw causal inferences, as other external influences could have contributed to the observed changes. Nevertheless, this design provides valuable insights into changes in adolescents’ vaccination knowledge, particularly in public health research where randomized controlled trials may not be feasible. Second, the follow-up period was relatively short, which may not fully capture the sustainability of the intervention’s effects over time. Despite this, the immediate feedback provided by the pre-post design allows for the assessment of the intervention’s preliminary effectiveness. Third, the study was conducted in selected high schools in a single geographical area, and the sample was predominantly female and Italian, which may limit generalizability to other regions or educational settings or more diverse populations. Fourth, self-reporting information has not been corroborated, and there is a possibility of a social desirability bias that could have contributed to over-estimation of responses. Moreover, anamnestic information on personal and parental chronic medical conditions was based on adolescents’ self-reports and not on clinically verified diagnoses. Consequently, these data may be subject to misunderstanding or incomplete knowledge of health status, which could have introduced some misclassification. However, participants did not provide identifying information, so they knew that their answers were completely anonymous, and this may have increased honesty. Fifth, students’ participation was voluntary and required parental consent, introducing potential selection bias that could have led to an overrepresentation of health-motivated students and an underrepresentation of those vaccine-hesitant, potentially inflating estimates of intervention effectiveness and limiting generalizability. Despite these limitations, this survey provides useful information for public health policymakers on the adolescents’ knowledge about vaccinations.

## 5. Conclusions

The findings of this study indicate that a school-based educational intervention may improve vaccination-related knowledge among high-school students. Although the present findings suggest that integrating structured educational activities in school settings may represent an effective strategy to enhance knowledge related to vaccination and support public health vaccination programs, further studies incorporating control groups, long-term follow-up, and students’ feedback are needed to confirm effectiveness.

## Figures and Tables

**Table 1:** Socio-demographic and anamnestic characteristics of the study population.

Characteristics		
***Socio-Demographic***		
	**Mean ± Standard Deviation (Range)**	
**Age, years (continuous)**	15.8 ± 1 (14–19)	
**Number of cohabitants (377) ^a^**	3.6 ± 1.2 (1–12)	
	**N**	**%**
**Gender (380) ^a^**		
Male	98	25.8
Female	282	74.2
**Nationality (378) ^a^**		
Italian	359	94.2
Foreign	22	5.8
**Mother with a university degree (371) ^a^**		
No	324	87.3
Yes	47	12.7
**Father with a university degree (364) ^a^**		
No	316	86.8
Yes	48	13.2
**Mother employed (357) ^a^**		
No	211	59.1
Yes	146	40.9
**Father employed (346) ^a^**		
No	27	7.8
Yes	319	92.2
***Anamnestic***		
**Chronic medical condition (386) ^a^**		
No	328	85
Yes	58	15
**Parents’ chronic medical condition (373) ^a^**		
No	295	79.1
Yes	78	20.9

^a^ Number of respondents for each item.

**Table 2:** Knowledge levels pre- and post-intervention with McNemar test results.

Knowledge	Pre-Intervention Correct	Post Intervention Correct	McNemar		
	N (%)	N (%)	Χ^2^	95% CI *	*p* Value
Q1. What is the immune system?	345 (89.6)	352 (93.1)	3.19	0.92–1.01	0.074
Q2. What is innate immunity?	98 (25.6)	150 (39.7)	26.58	0.52–0.75	<0.001
Q3. Which one of the following is an example of naturally acquired immunity?	224 (58)	226 (60)	0.29	0.88–1.07	0.593
Q4. How do vaccines work?	197 (51)	227 (60.2)	11.17	0.76–0.93	0.001
Q5. Which of the following is a vaccine advantage?	235 (61.2)	260 (69.1)	7.76	0.81–0.96	0.005
Q6. What is herd immunity?	277 (72.5)	296 (78.3)	4.94	0.86–0.99	0.026
Q7. Which of the following vaccinations are recommended for your age group?	143 (37.4)	230 (61.2)	57.78	0.55–0.71	<0.001

* CI = Confidence Interval.

**Table 3:** Results of the multivariate linear regression model.

Variable *	β	SE	t	*p*
Model. Profile of participants who achieved higher post-intervention test scores F (7, 309) = 19.08; R^2^ = 30.2%; adjusted R^2^ = 28.5%; *p* < 0.0001					
Higher pre-intervention test scores	0.47	0.05	8.28	<0.001
Willingness to participate in similar future interventions	0.62	0.21	2.96	0.003
Having the father employed	0.71	0.29	2.43	0.015
Having parents with chronic medical conditions	0.41	0.19	2.20	0.028
Foreign	−0.68	0.35	−1.93	0.055
Older	0.11	0.08	1.30	0.194
Lower number of cohabitants	−0.09	0.06	−1.40	0.164

* The following variables were deleted during the stepwise procedure: gender, type of school, being satisfied with the educational intervention, and considering the information provided as clear.

## Data Availability

The data presented in this study are available upon request from the corresponding author.

## Supplementary Materials

The following supporting information can be downloaded at https://www.mdpi.com/article/10.3390/vaccines14020153/s1, File S1: Questionnaire.

## Abbreviations

The following abbreviations are used in this manuscript:

dTaP-IPV	Diphtheria, tetanus, acellular pertussis, and inactivated polio vaccine
MenACWY	Meningococcal serogroup ACWY
HPV	Human Papillomavirus
VPDs	Vaccine-preventable diseases
