# Supplementary material for: Improving Vaccine Knowledge Among Adolescents: A Pre–Post School-Based Educational Intervention in Southern Italy
Source: Vaccines (Basel). 2026 Feb 4;14(2):153. doi: 10.3390/vaccines14020153 (PMC12944830; doi:10.3390/vaccines14020153)
Supplement: Supplementary file 1 [file vaccines-14-00153-s001.zip › vaccines-4098579-supplementary.pdf]

Socio-demographic and anamnestic characteristics

1. What gender do you identify as? □ Male □ Female □ Non-binary
2. How old were you on your last birthday? ________
3. What is your nationality? □ Italian □ Other, specify ________
4. Indicate the number of cohabitants: ________
5. What is your mother's highest educational level?
□ Primary school □ Middle school □ High school □ University degree
6. What is your father's highest educational level?
□ Primary school □ Middle school □ High school □ University degree
7. What is your mother's occupational status? □ Unemployed □ Employed, specify ________
8. What is your father's occupational status? □ Unemployed □ Employed, specify __________
9. Do you have any chronic medical conditions? □ No □ Yes, specify ________
10. Do your parents have any chronic medical conditions? □ No □ Yes, specify _________

Knowledge Related to Vaccination

11. What is the immune system?
- □ It is a shield that protects our body
- □ It is a system that self-regulates vital functions
- □ A system that makes a population immune
- □ A system that aids digestion

12. What is innate immunity?
- □ It is an immunity specific to each disease
- □ It is the first line of defence and is non-specific
- □ It is an immunity that occurs exclusively at birth
- □ It is an immunity that takes several days to activate

13. Which of the following is an example of naturally acquired immunity?
- □ Taking an antibiotic for an infection
- □ Recovering from a disease and becoming resistant to it
- □ Receiving a vaccine
- □ Washing hands frequently

14. How do vaccines work?
- □ They introduce live pathogens into the body to cause illness and strengthen the immune system
- □ They stimulate the immune system by exposing it to a weakened or inactive form of the pathogen, enabling future recognition
- □ They eliminate all viruses and bacteria in the body
- □ They provide immediate protection against any infection without the need for an immune response

15. Which of the following is a vaccine advantage?
- □ It protects only and exclusively the vaccinated individual
- □ It makes the immune system weaker over time
- □ It helps prevent the spread of diseases and protects those who cannot be vaccinated
- □ It prevents all types of infections, including those not covered by the vaccine

16. What is herd immunity?

□ When a group of people decides to get infected simultaneously

□ When a large part of the population is vaccinated, hindering the spread of a disease and protecting those who cannot be vaccinated

□ When everyone has previously contracted a specific disease at least once

□ When only children are vaccinated to protect adults

17. Which of the following vaccinations are recommended for your age group?

□ HPV, if not previously administered

□ Diphtheria, tetanus, pertussis, and polio

□ Meningococcus B

□ All of the above

Adolescents' Perception of the Intervention

18. Assign a score from 1 (not at all) to 5 (completely):

18a. How much are you satisfied with the educational intervention?

1 2 3 4 5

18b. How much do you consider the information provided clear?

1 2 3 4 5

18c. How much do you consider the acquired knowledge useful for making daily health-related decisions?

1 2 3 4 5

19. Do you need further information on vaccination? □ No □ Yes

20. If your school offered similar future interventions, would you be interested in participating? □ No □ I do not know □ Yes, if so, which topics would you be interested in? (please specify) ________
